# Cigarette smoke induces cell motility via platelet-activating factor accumulation in breast cancer cells: a potential mechanism for metastatic disease

**DOI:** 10.14814/phy2.12318

**Published:** 2015-03-22

**Authors:** Shannon Kispert, John Marentette, Jane McHowat

**Affiliations:** Department of Pathology, Saint Louis University School of MedicineSt. Louis, Missouri

**Keywords:** Breast cancer, cigarette smoke, motility, platelet-activating factor

## Abstract

Most cancer deaths are a result of metastasis rather than the primary tumor. Although cigarette smoking has been determined as a risk factor for several cancers, its role in metastasis has not been studied in detail. We propose that cigarette smoking contributes to metastatic disease via inhibition of breast cancer cell platelet-activating factor acetylhydrolase (PAF-AH), resulting in PAF accumulation and a subsequent increase in cell motility. We studied several breast cell lines, including immortalized mammary epithelial cells (MCF-10A), luminal A hormone positive MCF-7, basal-like triple negative MDA-MB-468, and claudin-low triple-negative highly metastatic MDA-MB-231 breast tumor cells. We exposed cells to cigarette smoke extract (CSE) for up to 48 h. CSE inhibited PAF-AH activity, increased PAF accumulation, and increased cell motility in MDA-MB-231 metastatic triple negative breast cancer cells. The calcium-independent phospholipase A_2_ (iPLA_2_) inhibitor, (*S*) bromoenol lactone ((*S*)*-*BEL) was used to prevent the accumulation of PAF and further prevented the increase in cell motility seen previously when cells were exposed to CSE. Thus, iPLA_2_ or PAF may represent a therapeutic target to manage metastatic disease, particularly in triple-negative breast cancer patients who smoke.

## Introduction

Cancer is a major cause of mortality worldwide, contributing to 7.5 million deaths annually. Breast cancer is the most common malignant disease affecting women in the Western world and is responsible for the most cancer deaths in women worldwide (Weigelt et al. 2005; Redig and McAllister 2013). The main cause of mortality does not lie in the primary tumor, but rather metastasis to distant sites. Once the primary tumor is identified, about 15% of breast cancer patients develop distant metastatic disease in 3 years, and remain at risk for metastasis decades later (Weigelt et al. 2005).

Despite metastasis being responsible for almost 90% of cancer-related deaths, there are many aspects of metastasis that remain undefined. During metastasis, a tumor cell detaches from its neighbor, mobilizes and invades surrounding tissue, undergoing intravasation into the lymph or blood vessels. This allows the tumor cell to relocate to distant sites via the bloodstream. Tumor cells then must extravasate from the bloodstream and proliferate in the secondary site (Chaffer and Weinberg 2011). The pivotal mechanism of metastatic disease is the autonomous ability of tumor cells to mobilize and eventually invade across impermeable tissues (Palmer et al. 2011). Therefore, investigation into possible stimulators of tumor cell invasion remains important.

There are many risk factors for cancer development and subsequent metastasis which include lifestyle choices such as poor diet, alcohol consumption, and cigarette smoking (Ligibel 2012). Several studies have linked cigarette smoke exposure with increased risk of breast cancer development and metastasis (Miller et al. 2007; Johnson et al. 2011; Luo et al. 2011; Di Cello et al. 2013). However, the mechanism by which cigarette smoke alters breast tumor cells and contributes to metastasis remains unclear (Di Cello et al. 2013).

An important mediator in cancer metastasis is platelet-activating factor (PAF), a potent inflammatory lipid mediator involved in many phases of cancer progression including tumor growth, metastasis, and angiogenesis (Bussolino et al. 1995; Camussi et al. 1996, 1997; Bussolati et al. 2000). Platelet-activating factor synthesis occurs following phospholipase A_2_ (PLA_2_)-catalyzed hydrolysis of membrane phospholipids to lysophospholipids and transacetylation by lysoPAF-acetyltransferase, using acetyl-CoA to form biologically active PAF (McHowat et al. 2001). Platelet-activating factor has been shown to trigger a variety of intracellular pathways including those involving tumor angiogenesis (Bussolati et al. 2000). The presence of PAF has been detected in breast cancer tissue and correlates with microvessel density (Bussolati et al. 2000). Specific breast cancer cell lines such as MDA-MB-231 have been shown to have higher PAF content which can elicit cell motility and proliferation (Bussolati et al. 2000). The PAF receptor (PAF-R) has been detected in various cell lines including breast tumor cells. We have shown previously that MDA-MB-231, MDA-MB-468, and MCF-7 cell lines all express the PAF receptor and MDA-MB-231 triple negative cells increased PAF-R expression in response to CSE exposure. Normal mammary epithelial cell line MCF-10A exhibited no PAF-R expression even after 48 h of CSE exposure (Kispert et al. 2014).

We have shown that cigarette smoke inhibits endothelial cell PAF-AH, the enzyme responsible for PAF hydrolysis and inactivation (Sharma et al. 2012). PAF-AH inhibition led to the enhanced PAF accumulation and increased polymorphonuclear leukocyte (PMN) adherence in human microvascular lung endothelial cells (HMVEC-L) (Sharma et al. 2012). We have also recently demonstrated cigarette smoke exposure increases adherence of MDA-MB-231 breast tumor cells to the lung endothelium (Kispert et al. 2014). This inhibition could be abrogated by pretreatment of MDA-MB-231 cells with an iPLA_2_*β* inhibitor, (S)-bromoenol lactone (*S*)*-*BEL, which inhibits PAF production (Kispert et al. 2014). These experiments introduced the novel idea that cigarette smoke could affect PAF accumulation in breast cancer cells and possibly effect motility and metastasis.

Here, we present effects of cigarette smoke extract on PAF accumulation and cell motility in breast cancer cells. Cell lines used include the highly invasive triple-negative MDA-MB-231 human breast cancer cell line and the triple negative, less invasive MDA-MB-468. Triple-negative breast cells are deficient in estrogen and progesterone receptors and these cancers are generally more aggressive. Less metastatic cell lines used include hormone positive nonmetastatic MCF-7 human breast cancer cells which express estrogen and progesterone receptors and the normal mammary epithelial cell line MCF-10A (Neve et al. 2006; Kao et al. 2009). We found that cigarette smoke decreases PAF-AH activity in breast cancer cells and consequently increases PAF accumulation. PAF receptor expression in breast cancer cell lines is also increased with CSE exposure. In cell motility measurements, breast cancer cells incubated with CSE showed increased motility over those incubated with untreated medium.

## Materials and Methods

### Materials

MDA-MB-231, MDA-MB-468, MCF-7, and MCF-10A cells were obtained from American Type Culture Collection (ATCC) Manassas, VA. Cigarette smoke extract (CSE) was obtained from Murty Pharmaceuticals (Lexington, KY). Cigarette smoke extract was prepared by smoking University of Kentucky's Research Cigarette and extracting total particulate matter with DMSO (Roemer et al. 2012). [^3^H] acetic acid, sodium salt and hexadecyl-2-acetyl-*sn*-glyceryl-2-phosphorylcholine, 1-*O*-[acetyl-^3^H(N)] were obtained from PerkinElmer (Boston, MA). (*S*)-BEL, (*R*)*-*BEL, and all other chemicals were obtained from Sigma Chemical (St. Louis, MO).

### Cell culture

MDA-MB-231, MDA-MB-468, MCF-7, and MCF-10A were grown to confluence in DMEM or MEGM supplemented with growth factors (Lonza, Walkersvile, MD) and incubated at 37°C in an atmosphere of 95% O_2_–5% CO_2_. Cells were passaged at 1:3 ratio using trypsin/EGTA. All experiments were conducted with cell monolayers. Some cells were treated with 20 *μ*g/mL of CSE. This concentration most accurately reflects representation in human serum, does not affect cell viability, and results in PAF accumulation (Kier et al. 1974).

### PAF-AH activity

Cells were grown to confluence in 35-mm dishes, removed in 1.2 mmol/L Ca^2+^-HEPES buffer, and sonicated on ice. Cellular protein (25 *μ*g) was incubated with 0.1 mmol/L [^3^H]acetyl-PAF (10 mCi/mmol) for 30 min at 37°C. The reaction was stopped by adding 50 *μ*L of 10 mol/L acetic acid and 1.5 mL of 0.1 mol/L sodium acetate. Released [^3^H]acetic acid was isolated by passing the reaction mixture through a C_18_ gel cartridge (Baker Chemical, Phillipsburg, NJ), and radioactivity was measured using a liquid scintillation counter (Sharma et al. 2012).

### PAF accumulation

Cells grown in 35-mm dishes were washed twice with Hanks' balanced salts solution (HBSS) and incubated with 10 *μ*Ci [^3^H]acetic acid per well for 20 min. After experimental conditions, cellular lipids were extracted using the method of Bligh and Dyer (Bligh and Dyer 1959). The chloroform layer was concentrated under N_2_, resuspended in 9:1 CHCl_3_-MeOH, applied to a Silica Gel 60 TLC plate, and developed in chloroform–methanol–acetic acid–water (50:25:8:4 vol/vol/vol/vol). The region corresponding to [^3^H]PAF was scraped, and radioactivity was quantified using liquid scintillation spectrometry. Loss of PAF during extraction and chromatography was corrected by adding a known amount of [^14^C]PAF as an internal standard (Sharma et al. 2012).

### Cell motility

Cell motility was determined using the electric cell-substrate impedance sensing (ECIS) system (Applied Biophysics, Troy, NY) as described previously (Keese et al. 2004). Cells were grown on ECIS electrode arrays (8W1E). The impedance fluctuations of cell attachment and spread were continuously monitored. An alternating current of 1 *μ*A at 4 kHz was applied between a small sensing electrode (250-*μ*m diameter) and a relatively large counter electrode. At confluence, CSE (20 *μ*g/mL) was added to the culture medium. Experiments were conducted up to 2 days after CSE introduction. Wounding of cells was achieved using a 6V signal at 45 kHz for a duration of 30 sec. Application of this field results in a rapid drop in the impedance of cell layers due to the death of the cells on the electrode. Impedance increases as cells migrate from the perimeter of the electrode inward to replace the wounded cells.

### Statistical analysis

All studies were repeated with at least three separate cell cultures. Data were analyzed using Student's *t*-test or one-way analysis of variance followed by post hoc analysis using Dunnett's test. Differences were regarded as significant at *P* < 0.05 and highly significant at *P* < 0.01. Data are mean ± SE.

## Results

### Breast cancer cell PAF-AH activity is inhibited and PAF accumulates with exposure to CSE

**Figure 1:**
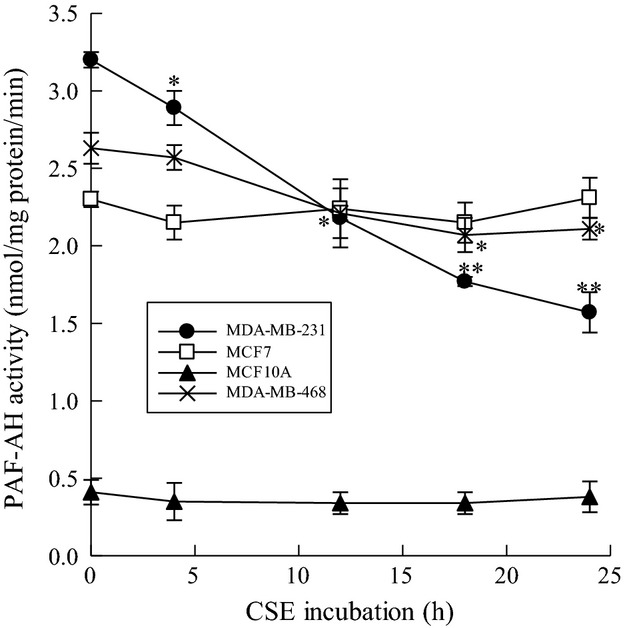
CSE incubation inhibits PAF-AH activity in triple-negative breast cancer cell lines. Platelet-activating factor acetyl hydrolase (PAF-AH) activity in MDA-MB-231(•), MCF-7(□), MDA-MB-468 (X), and MCF-10A(▴) cells incubated with CSE (20 *μ*g/mL) for up to 24 h. Values shown are mean ± SEM for eight different cell cultures. *P < 0.05, **P < 0.01 when compared with controls.

We incubated MDA-MB-231, MCF-7, MCF-10A, and MDA-MB-468 cells with CSE (20 *μ*g/mL) for increasing time intervals and measured PAF-AH activity (Fig.1). Significant inhibition of PAF-AH activity was observed with CSE incubation in the more invasive breast cancer cell lines MDA-MB-231 and MDA-MB-468. The decrease in PAF-AH activity was time dependent with CSE incubation (Fig.1). However, inhibition of PAF-AH activity was not evident in the nonmetastatic cell line MCF-7 and normal mammary epithelial cell line MCF-10A. As shown in Figure1, PAF-AH activity was much lower in MCF-10A cells when compared to all breast cancer cell lines tested.

**Figure 2:**
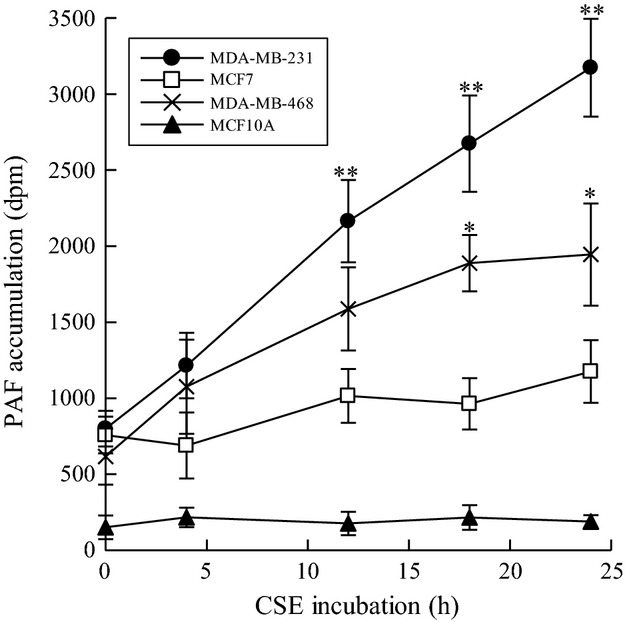
CSE incubation increases PAF accumulation in triple-negative breast cancer cell lines. Platelet-activating factor (PAF) accumulation in MDA-MB-231(•), MCF-7(□), MDA-MB-468 (X), and MCF-10A(▴) cells incubated with CSE (20 *μ*g/mL) for up to 24 h. Values shown are mean ± SEM for eight different cell cultures. *P < 0.05, **P < 0.01 when compared with controls.

Since PAF-AH activity is inhibited by CSE in metastatic breast cancer cells, we measured PAF accumulation in the same cells. We incubated MDA-MB-231, MCF-7, MCF-10A, and MDA-MB-468 breast cells with CSE (20 *μ*g/mL) at increasing time points and measured PAF accumulation (Fig.2). We observed significant PAF accumulation in MDA-MB-231 and MDA-MB-468 cells when compared to MCF-7 or MCF-10A cells (Fig.2). The time course of PAF accumulation (Fig.2) coincided with CSE-dependent PAF-AH inhibition (Fig.1). In normal mammary epithelial MCF-10A cells, we observed much lower PAF content than that measured in breast cancer cell lines that was unaffected by CSE exposure at any time point measured.

### iPLA_2_ inhibitors can mediate the effects of CSE on PAF accumulation

**Figure 3:**
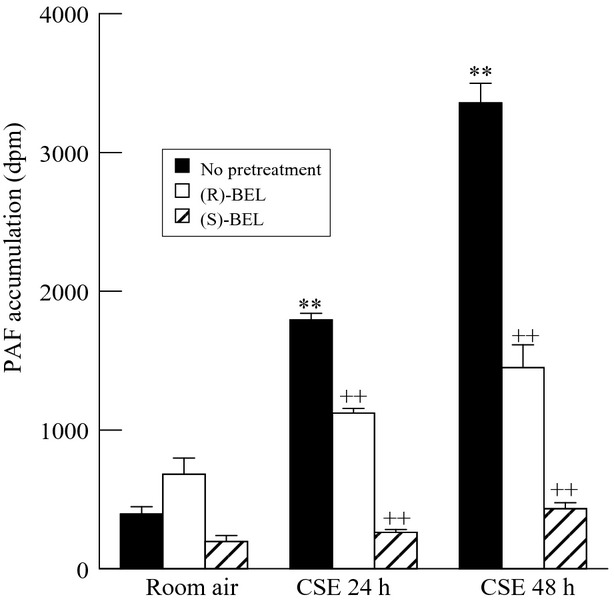
PAF accumulation in MDA-MB-231 cells pretreated with iPLA_2_ inhibitors. Cells were pretreated with 0.5 *μ*mol/L (*R*)*-*bromoenol lactone (BEL) or (*S*)*-*BEL (10 min) prior to the addition of CSE (20 *μ*g/mL, 24-48 h) and both remained throughout the culture. Values shown are mean ± SEM for six separate cell cultures. **P < 0.01 when compared to control (media) PAF content. ^++^*P* < 0.01 when comparing values in the presence or absence of BEL.

Two iPLA_2_ isoforms (iPLA_2_*β* and iPLA_2_*γ*) are predominant in endothelial cells (Sharma et al. 2011). The (*S*) enantiomer of the compound BEL preferentially inhibits iPLA_2_*β* and (*R*)-BEL inhibits iPLA_2_*γ* (Jenkins et al. 2002). In vitro studies have shown that iPLA_2_*β* is the key enzyme involved in PAF accumulation (Rastogi and McHowat 2009). We utilized iPLA_2_ inhibitors to monitor changes in PAF accumulation in response to CSE incubation. The cells were treated with (*S*)*-*BEL and (*R*)-BEL prior to incubation with CSE. Metastatic breast cancer cells (MDA-MB-231) showed a significant increase in PAF accumulation when treated with CSE for 24 or 48 h (Fig.3). This was significantly inhibited by pretreatment with (*S*)-BEL at both 24 and 48 h of CSE exposure. (*R*)-BEL had minimal effects on CSE-induced PAF accumulation at both 24 and 48 h of exposure (Fig.3). These data indicate that MDA-MB-231 cell PAF accumulation is dependent upon iPLA_2_*β* activity.

### CSE and iPLA_2_ inhibitors can mediate the effects of CSE on cell motility

**Figure 4:**
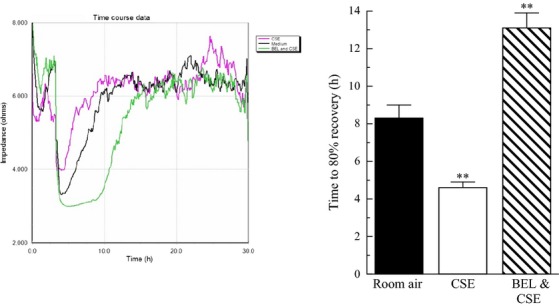
CSE incubation increases breast cancer cell motility. MDA-MB-231 cells were exposed to CSE (pink), media only (black), or CSE and (*S*)-BEL (green) before and after wounding and measured by ECIS. Bar graph depicts time to 80% recovery for MDA-MB-231 cells exposed to media only (black), CSE (white), and BEL/CSE (striped).

To investigate the effects of cigarette smoke incubation and subsequent PAF accumulation on breast cancer cell motility and proliferation, we utilized the ECIS system to measure impedance across MDA-MB-231 and MCF-7 cell monolayers cultured on gold electrodes. After wounding, we measured cell motility in real time in cell lines exposed to CSE or left untreated (Fig.4). The rate of wound healing, as measured by impedance values returning to prewounding values, was increased in highly metastatic MDA-MB-231 cells that were incubated with CSE (pink) when compared to those cultured in untreated media only (black) (Fig.4). To determine whether increased cell motility in the presence of CSE was mediated by iPLA_2_*β* in MDA-MB-231 cells, we treated cells with (*S*)*-*BEL (Fig.4). MDA-MB-231 cells treated with CSE (pink) demonstrated increased cell motility over those with media alone (black); however, when cells are pretreated with (*S*)*-*BEL (green), they exhibit decreased motility when compared to media alone or those incubated with CSE (black) (Fig.4). The graph in Figure4 reflects mean data from the ECIS experiments demonstrating the overall decrease in time to 80% recovery when cells are cultured with CSE and the increase in time to 80% recovery when (*S*)*-*BEL is added to the culture. This suggests that the inhibition of iPLA_2_*β* and its subsequent reduction of CSE-induced PAF accumulation can abrogate the effects on cell motility.

**Figure 5:**
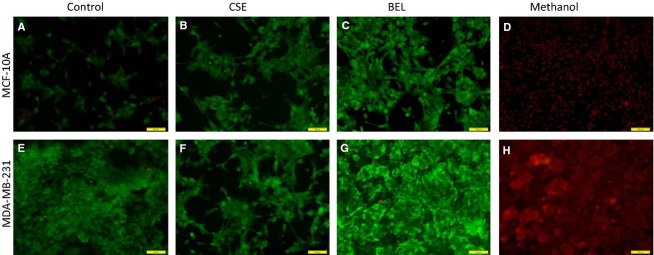
Effects of CSE and (*S*)*-*BEL on cell viability. MDA-MB-231 and MCF-10A cell viability measured using the LIVE/DEAD viability/cytotoxicity assay kit (Invitrogen). Cells were left untreated (A, E)*,* incubated with CSE (20 *μ*g/mL) (B, F) or (*S*)*-*BEL (0.5 *μ*mol/L) (C, G) for 24 h. Cells were treated with methanol (30 min) as a positive control for cell death (D, H).

To determine whether the addition of CSE and (*S*)*-*BEL had any effect on cell viability for the previous experiments, we utilized the LIVE/DEAD viability/cytotoxicity assay kit (Invitrogen, Grand Island, NY, USA) (Fig.5). Cells were left untreated (A, E)*,* incubated with CSE (20 *μ*g/mL) (B, F) or (*S*)*-*BEL (C, G) for 24 h. No difference in viability was observed when comparing experimental groups to controls. Cells were treated with methanol (30 min) as a positive control for cell death (D, H).

## Discussion

Platelet-activating factor is an inflammatory mediator implicated in the pathology of several human diseases that include diabetes, hypertension, and cancer (Tjoelker and Stafforini 2000). Platelet-activating factor was first associated with cigarette smoke-induced tissue injury when smokers displayed increased plasma PAF concentration (Imaizumi 1991). Platelet-activating factor has more recently been implicated in many phases of cancer progression involving tumor growth, metastasis, and angiogenesis (Bussolati et al. 2000). PAF has been found in breast cancer tissue and correlates with microvessel density (Bussolati et al. 2000). Some metastatic cell lines are shown to have higher PAF content which can subsequently elicit cell motility and proliferation.

We have recently shown that cigarette smoke inhibits PAF-AH, the enzyme responsible for PAF inactivation, leading to PAF accumulation in lung endothelial cells (Sharma et al. 2012). In this study, we observe similar responses in triple-negative breast cancer cell lines. MDA-MB-231 and MDA-MB-468 show inhibited PAF-AH activity and increased PAF accumulation with CSE exposure. The hormone positive, less metastatic MCF-7 cells exhibit no decreases in PAF-AH activity and only minimal increases in PAF accumulation. Immortalized normal mammary epithelial cells exhibit minimal PAF-AH activity and PAF content, with neither being influenced by CSE exposure.

PAF synthesis occurs following phospholipase A_2_ (PLA_2_) catalyzed hydrolysis of membrane phospholipids to form 1-O-acyl-sn-glycero-3-phosphocholine (lysoPAF). LysoPAF is subsequently transacetylated by lysoPAF-acetyltransferase using acetyl-CoA to form biologically active PAF. Metastatic breast cancer cells, MDA-MB-231, show a significant increase in PAF accumulation when treated with CSE for 24 h (Fig.1). This increased accumulation is significantly reduced by pretreatment with (*S*)-BEL (Fig.3). (*R*)-BEL produces no significant reduction in PAF accumulation (Fig.3). These data indicate that inhibitors of iPLA_2_*β* to block PAF synthesis are sufficient to ameliorate PAF accumulation, even in the setting of PAF-AH inhibition.

Cigarette smoke extract induced PAF accumulation could promote metastasis by several mechanisms, including increased tumor cell motility and invasiveness in breast tissue. Enhanced cell motility is characteristic of tumor cells in vitro and is an essential step in metastasis (Wang et al. 2005). Using ECIS, we observed proliferation and/or cell motility in MDA-MB-231 and MCF-7 breast cells exposed to CSE and untreated media. Triple-negative MDA-MB-231 cells incubated with CSE exhibited higher motility in comparison with those cultured in media alone. Increased wound healing in the presence of CSE was blocked when cells were pretreated with (*S*)*-*BEL suggesting the involvement of iPLA_2_*β* and PAF production in cell motility/proliferation. However, the low invasive MCF-7 cells do not exhibit increased motility in response to incubation with CSE. Thus, the lack of CSE-induced cell motility correlates with the absence of increased PAF accumulation in MCF-7 cells. This suggests that cigarette smoke exposure could cause increased motility or cell proliferation preferentially in triple-negative, highly metastatic tumors. Triple-negative breast cancer is associated with a poor prognosis and lack of specific targeted therapies. Tumor resistance to chemotherapy is an important clinical problem. A recent study suggests that PAF receptor agonists inhibit chemotherapy effectiveness (Sahu et al. 2014). Our data demonstrate that triple-negative, highly invasive breast cancer cells show increased PAF production when compared to less invasive breast cancer and mammary epithelial cells, which may represent a mechanism whereby this type of breast cancer is particularly difficult to treat.

In this study, we have studied the effect of short-term CSE exposure. However, we cannot rule out that longer exposure to cigarette smoke, such as that experienced in a breast cancer patient who smokes would not increase the motility of less invasive breast cancer cell lines such as MCF-7 or even noncancerous cell lines such as MCF-10A.

These data provide a novel insight into how smoking can affect tumor cells via mediation of PAF. The inflammatory mediator PAF is known to cause increased adherence of tumor cells to the endothelium, increased angiogenesis, and enhanced oncogene expression (Tripathi et al. 1991; Mannori et al. 2000). PAF is increased in various pathologies such as human breast, colorectal, and lung cancers (Montrucchio et al. 1998; Denizot et al. 2001, 2005). Our data provide an underlying mechanism whereby PAF may accumulate and mediate tumor progression via PAF-AH inhibition in smokers.

In conclusion, we show here for the first time that CSE exposure to breast cancer cells leads to the inhibition of PAF-AH and the accumulation of PAF. In attempting to mitigate the effects of CSE on PAF accumulation, we used an iPLA_2_*β* inhibitor, (*S*)*-*BEL, and blocked PAF accumulation completely. When observing the effects of CSE on tumor progression, we show that triple-negative MDA-MB-231 breast cancer cells exposed to CSE exhibit higher motility in comparison with those treated with media alone. Together these data provide a mechanistic framework for the observed effects of cigarette smoke exposure on breast tumor cells via the inflammatory mediator PAF.

## Conflict of Interest

None declared.
